# Participation Strategies of Parents of Children with Neurodevelopmental Disorders: An Exploratory Study

**DOI:** 10.3390/children11020192

**Published:** 2024-02-03

**Authors:** Hiroyasu Shiozu, Daisuke Kimura, Ryoichiro Iwanaga, Shigeki Kurasawa

**Affiliations:** 1 Department of Occupational Therapy, College of Life and Health Sciences, Chubu University, Kasugai 487-0027, Japan; 2 Department of Occupational Therapy, Nagoya Woman’s University, Nagoya 467-8610, Japan; dkimura@nagoya-wu.ac.jp; 3 Department of Occupational Therapy Sciences, Nagasaki University, Nagasaki 852-8131, Japan; iwanagar@nagasaki-u.ac.jp; 4 Department of Occupational Therapy, School of Health Sciences, Fukushima Medical University, Fukushima 960-1295, Japan; kurasawa@fmu.ac.jp

**Keywords:** participation, strategy, neurodevelopmental disorder, elementary children, parent

## Abstract

Strategies are critical to promote child participation in important life activities. This study analyzed the participation strategies of the parents of children with neurodevelopmental disorders. Ninety-two Japanese elementary children with neurodevelopmental disorders and their parents were recruited. The parents completed the Participation and Environment Measure for Children and Youth (PEM-CY) questionnaire. Strategy text data obtained from the PEM-CY were analyzed with the co-occurrence network and correspondence analyses. The co-occurrence network analysis showed that the commonality of strategies to enable participation at home, school, and community settings was able to explain the child’s characteristics when involved in each setting. The correspondence analysis also suggested the need for specific strategies in each setting. The importance of strategies to improve the attitudinal environment and promote the participation of children with neurodevelopmental disorders was evident. Reducing stigma is important in all environments, especially in the public sphere. In addition, specific strategies are needed in each setting, suggesting the importance of context-specific approaches.

## 1. Introduction

Child and youth participation in crucial life situations at home, school, work, and in the community is an important outcome for families and children. Furthermore, it is a research priority in pediatric rehabilitation [1]. In recent years, approaches focused on the participation of children with cerebral palsy revealed positive results. Pathways and Resources for Engagement and Participation (PREP) is an example of one such approach. PREP is an evidence-based approach to occupational therapy that focuses on enhancing participation through modifying the environment [2]. Initially, health promotion was defined as “health promotion is the process of enabling people to increase control over, and to improve their health”, and in order “to reach a state of complete physical, mental and social well-being, an individual or group must be able to identify and to realize aspirations, to satisfy needs, and to change or cope with the environment” in the Ottawa Charter for Health Promotion [3]. Modifying the environment in PREP is a reasonable approach from a health promotion perspective. Furthermore, one of the key techniques of PREP is “coaching”. Coaching is one intervention technique that enables client participation. Other approaches that include coaching are Cognitive Orientation to Daily Occupational Performance (CO-OP) [4], Occupation Performance Coaching (OPC) [5], and Partnering for Change (P4C) [6].

However, evidence on the value of improving children’s participation in pediatric occupational therapy, particularly the participation of children with neurodevelopmental disorders (NDDs), is insufficient, and further research is needed [7]. One of the key elements in these approaches, including coaching, is how to work with the client to discover effective strategies. From this perspective, we must analyze the client’s participation strategies to achieve their enhanced involvement in these activities.

In Japan, research on the participation of children with NDDs is a priority issue. Japan’s Ministry of Education, Culture, Sports, Science, and Technology survey found that an estimated 8.8% or about 1 in 11 elementary and junior high school students may have NDDs and need special support but are enrolled in regular classes [8]. This figure represents an increase of 2.5% from a previous survey conducted ten years ago. Subsequently, the enhancement of support in schools has started receiving scholarly attention. In 2022, the number of truant students among long-term absentees in elementary and junior high schools was 299,048 (up from 244,940 in the previous year), and the number of truant students per 1000 students was 31.7 (increased from 25.7 in the previous year). The number of children not attending school increased for 10 consecutive years, reaching a record high in 2022 [9]. A 20-year longitudinal birth cohort study in Japan revealed that 23.5% of children with autism spectrum disorder (ASD) aged less than seven refused to attend school between elementary and high school [9]. This rate was 30.6% among children with ASD without intellectual disabilities [9]. Moreover, the psychosocial outcomes were “poor” for 25.6% and “very poor” for 4.8% (based on a five-level classification ranging from very good to very poor) [10]. The most common reason for school refusal among children with ASD has been found to be bullying [11]. Such reasons are influenced by social factors, one of which is stigma. Stigma is influenced by culture; for instance, stigma toward ASD has been found to be higher among Japanese college students than among college students in the United States [12].

Considering this social context of Japan, there is a need to further support the participation of children with NDDs. However, effective support methods have not been established, and there is insufficient research on the state of their participation in their home, school, and community. Furthermore, for participation to arise from the dynamics of various factors, it is necessary to analyze participation from the perspective of the clients themselves.

Therefore, this study aimed to analyze parents’ strategies to enable the participation of children with NDDs using the Participation and Environment Measure for Children and Youth (PEM-CY). This endeavor allows us to understand the state of the participation of these children in Japan, which has remained underexplored. It also sheds light on participation strategies from clients’ perspectives and allows us to support their participation in the future.

## 2. Materials and Methods

### 2.1. Study Design

This nonexperimental, single-group, cross-sectional study was conducted from April to June 2023. It was also an exploratory study that analyzed highly individualized client participation strategies derived from textual data. In this way, we explored the commonalities and heterogeneities of participation strategies in each setting (home, school, and community) and the factors that constrain participation derived from these strategies.

### 2.2. Participants

We recruited the parents of children with NDDs who were undergoing rehabilitation at a pediatric rehabilitation hospital in Japan. The inclusion criteria were as follows: (1) the child had been diagnosed according to the Diagnostic and Statistical Manual of Mental Disorders-5 (DSM-5) system by a pediatrician or child psychiatrist, and (2) the child was in elementary school and aged between 6 and 12 years (enrolled in the first through sixth grades). Four types of elementary classes and schools exist in Japan: regular classes, special support services in resource rooms, special support classes, and special support schools. All these educational settings were included.

### 2.3. Measures

#### Participation and Environment Measure for Children and Youth (PEM-CY)

The PEM-CY is a parent-reported measure divided into the following two sections: participation and environment. These sections can evaluate the participation of children and youth with and without disabilities and the environmental factors that affect their participation across three settings: home, school, and community [13]. The participation section measures three participation dimensions in each setting: frequency, the extent of involvement, and desire for change. The PEM-CY home and community participation scales comprise 10 activity items each, and the school participation scale comprises five activity items. The environmental section measures the environmental support and barriers that influence the children’s participation in each setting. The home subsection comprises 12 items, the school comprises 17 items, and the community comprises 16 items. This study used the overall environmental support score, which was the sum of all section scores. This sum was then converted to percentages. Furthermore, for each setting, parents could enter up to three strategies for encouraging their child’s participation. Regarding the psychometric properties of the PEM-CY, its internal consistency has been found to be moderate to good (0.59 and above) across different scales. Its test–retest reliability has also been found to be moderate to good (0.58 and above) for a 1- to 4-week period [14].

The primary analysis conducted in this study was to examine the textual data collected on the strategies used by parents to encourage participation in each setting. For reference, we compared the scores obtained for each setting with the data in the manual.

### 2.4. Procedure

The participants were given a written and verbal explanation of the study, informed consent, and the questionnaire’s content by their therapists. Subsequently, they were asked to complete the PEM-CY and Kid- & Kiddo-KINDL Parents’ Version (KINDL) questionnaires if they agreed to participate in the study. Given the impact of participation status on psychosocial outcomes in children with NDDs [10], KINDL was used to determine the quality of life of the participants. The questionnaires were filled out in person with paper and pen when they visited the hospital. Participants were also informed that their participation or non-participation in the study would not affect any rehabilitation interventions. For participants who responded, their child’s demographics, age, sex, class affiliation, and diagnosis were determined based on hospital records.

### 2.5. Statistical Analyses

This study used an econometric text analysis to investigate the relationships among the strategies used for engaging children with NDDs at home, school, and in the community. Econometric text analysis organizes or analyzes text-type data using quantitative methods to conduct content analysis. The statistical method we used was KH Coder 3 (available for download at https://khcoder.net/ (accessed on 1 December 2023)) [15,16]. The co-occurrence network and correspondence analyses were selected based on multivariate analysis. We selected analytical methods because they are generated directly from the data, which minimizes researchers’ preconceptions and makes them suitable for discovering patterns in textual data. The co-occurrence network analysis was selected because it is used to discover related terms. Meanwhile, correspondence analysis was employed because it made it possible to interpret relationships between categories by condensing the data into a two-dimensional space and visualizing it.

The co-occurrence network depicts a network of words with similar occurrence patterns (i.e., words with a strong co-occurrence). Since the line connections indicate the co-occurrence of words, they are easy to visualize. This study created a network of words, external variables, and headings to find words characteristic of each part of the text. Jaccard coefficients were calculated for all combinations of analyzed words to measure the strength of co-occurrence. The area of the circle was proportional to the number of occurrences of the word.

The correspondence analysis shows the results using the extracted words in a two-dimensional scatter plot. It is suitable for dividing the data into several parts and examining the characteristics of each part. This time, a bubble plot was selected, where the words appearing more frequently were drawn in larger circles. Words near the origin (0, 0) showed no differences, while words far from the origin differed more markedly.

### 2.6. Ethical Considerations

All subjects gave their informed consent for inclusion before they participated in this study. This study was conducted in accordance with the Declaration of Helsinki, and the data collection procedure and research design were approved by the Ethics Committee of Chubu University (Number: 20220095).

## 3. Results

### 3.1. Participation

Initially, 137 parents with elementary school-aged children with NDDs who met the relevant criteria were invited to participate, with 92 parents finally included. Table 1 displays the children’s demographics. More than 70% of the children were male, with a mean age of 7.6 (SD 1.6) years. Around 39% of the children attended regular classes, with the rest attending classes/schools where special support was provided. The most common NDD diagnoses were ASD (30%), followed by the developmental coordination disorder (24%), intellectual disorder (23%), specific learning disorder (15%), and attention-deficit/hyperactivity disorder (8%). All participating parents were female. The average age of the participating parents was 40.7 (SD 4.0) years.

In addition, the mean KINDL total score in this study was 69.3 (SD 10.6), which is lower than the mean score for Japanese elementary schoolers without disabilities (Note: The average score for Japanese children without disabilities is 75.3 (SD 10.2)) [17].

### 3.2. PEM-CY Score

The participation status of the 92 children is described (Table 2). The score for frequency was 5.58 with (SD 0.85) points for the home setting, 3.97 (SD 1.53) points for the school setting, and 1.97 (SD 0.96) points for the community setting. The score for involvement was 3.90 (SD 0.70) points for the home setting, 3.51 (SD 1.20) for the school setting, and 3.18 (SD 1.20) for the community setting. The score for the desire for change was 64 (SD 28) % for the home setting, 61 (SD 36)% for the school setting, and 56 (SD 31)% for the community setting. The score for environmental support was 80 (SD 12)% for the home setting, 84 (SD 12)% for the school setting, and 81 (SD 12)% for the community setting.

Compared to the data in the manual [13], participation scores were lower than those for children (<12 ages) without disabilities in Canada and the United States on most items. Only the Home-Involvement score was slightly higher in this study (This study’s data: 3.90 (SD 0.70) points, comparative data: 3.89 (SD 0.51) points).

### 3.3. Participation Strategy

The results of the analysis show that the total number of words extracted (used) was 5576 (2615), and the number of different words (used) was 991 (811). The top 10 most frequently occurring words were “children” (62), “school” (46), “participation” (37), “teacher” (36), “communicate” (35), “activity” (33), “friend” (33), “together” (27), “community” (27), and “communication” (26).

**Figure 1:**
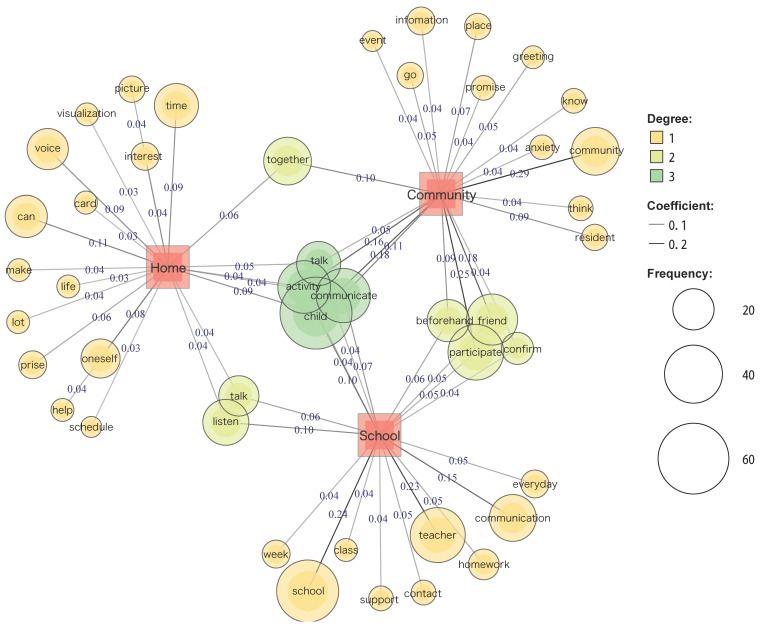
This figure illustrates the co-occurrence network analysis of participation strategies. The red boxes represent each PEM-CY setting. The orange circle (Degree 1) represents the specific word for each setting. The light green circle (Degree 2) represents the common word in the two settings. The green circle (Degree 3) represents the common word in all settings. The line width represents Jaccard coefficients, and the circle size represents word frequency.

Figure 1 shows the words with the strongest degree of co-occurrence in each of the three domains. In the home, school, and community settings, “child”, “communicate”, “activity”, and “talk” were strongly associated with each other. “Listening” and “talking” were strongly associated with the home and school settings. In the home and community settings, “together” showed strong common associations. In the school and community settings, “participation”, “friend”, “beforehand”, and “confirm” had strong common associations.

**Figure 2:**
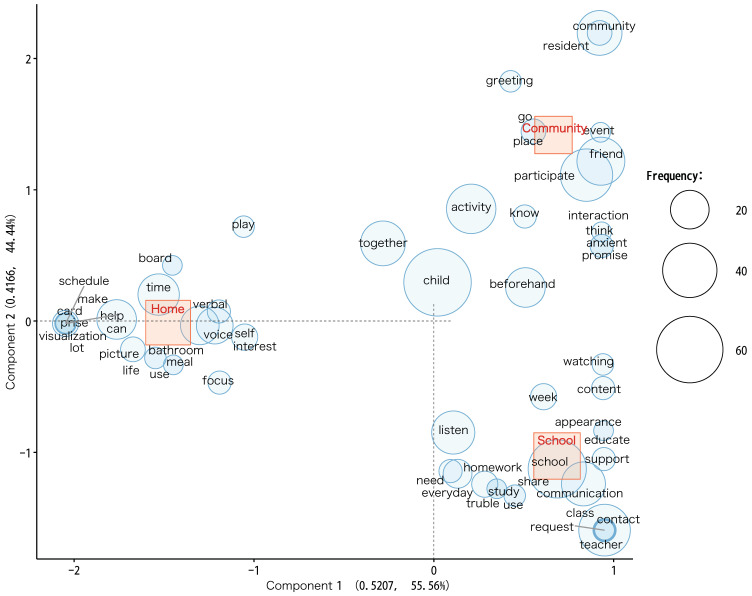
This figure illustrates the correspondence analysis of participation strategies. The red boxes represent each PEM-CY setting. Blue squares represent word position and frequency. Each setting is far from the origin, indicating a difference. However, in the first component, the results show a split between school/community and home. The component 1 and 2 axes in this chart are operationally set to visualize the main variations within the dataset. Note that these axes do not directly represent specific concepts but provide a foundation for understanding patterns and relationships in the data.

Figure 2 shows the relationship between the extracted words in the three regions regarding distance. The negative position of the first component is assigned to the home setting, and the positive position of the first component is assigned to the school and community settings. The negative position of the second component is assigned to the school setting, the positive position of the second component to the community setting, and the median position of the second component to the home setting. Each setting is markedly distant from its origin, indicating a substantive difference. However, the distribution of the positive position of the first component between the school and the community settings indicates a relationship between them. The eigenvalue of the first component was 0.5207, and the contribution was 55.56%. In contrast, the eigenvalue of the second component was 0.4166, and its contribution was 44.44%. The words near the origin were “child”, “together”, “activity”, and “activity”.

## 4. Discussion

Co-occurrence networks analyzed participation strategies. In the home, school, and community settings, “child”, “communicate”, “activity”, and “talk” were strongly associated with each other. In other words, we found that strategies involving discussion and communication concerning children’s characteristics were commonly used to engage them in activities in each setting. Regarding the actual strategy data, parents used strategies to explain their children’s characteristics and behaviors to friends, teachers, and community members to raise their awareness and increase their understanding. We believe that this strategy is used to reduce the stigma that affects participation. In fact, the raw data are littered with stigma-related strategies, such as “I often communicate with community members and parents of school classmates to make them aware of my child’s characteristics”, “(Parents) communicate a lot with elementary school teachers”, and “I try to communicate well with the parents of my children’s classmates.” Thus, it reveals the existence of stigma. Four types of stigmata have been identified [18]. They are self-stigma, stigma by association, public stigma, and structural stigma. Self-stigma occurs when people are aware of the negative stereotypes of others, agree with them, and turn them against themselves [18]. Stigma by association refers to the attribution of negative stereotypes and discrimination that are directed against family members or others [18]. Public stigma refers to stereotypes, negative attitudes, and discrimination against people with health conditions in society [18]. Structural stigma refers to policies and practices that work to the disadvantage of a stigmatized group, whether intentionally or unintentionally.

Among these are participation strategies against public stigma (also referred to as interpersonal stigma). A common way to understand public stigma is to identify three separate but related components: knowledge (ignorance), attitudes (prejudice), and behaviors (discrimination) [19]. In fact, according to a public opinion survey on people with disabilities conducted by Japan’s Cabinet Office in 2022, 88.5% of the respondents answered that they “think there is” discrimination and prejudice based on disability [20]. In addition, among those who responded that they “think there is” discrimination and prejudice based on disability, 40.4% answered that they “think that discrimination and prejudice against people with disabilities have not improved” compared to five years ago [20]. The stigma and exclusion of children with NDDs and their parents may be related to and increase their difficulties in community participation. In a literature review based on the stigma of the parents of children with ASD, most parents declared to be particularly susceptible to stigma in school environments and community settings [21]. We believe that our results share the background with the data of this study.

On the other hand, the view is that “hermeneutical injustice [22] “exists in the client’s perception”. Hermeneutical injustices occur “when a gap in collective interpretive resources puts someone at an unfair disadvantage when it comes to making sense of their social experiences” [22]. For example, parents limit their participation because they perceive their children’s behavior as disruptive or bothersome to those around them. Indeed, a study has found that mothers of children with disabilities experience negative judgments and social isolation [23]. This suggests that, in order to promote the participation of children with NDDs, there is a need not only to reduce stigma but also to support parents in changing their perceptions. In recent years, family-centered services (FCSs) [24] have evolved significantly, reflecting a shift towards more inclusive and holistic approaches in childcare and support. FCS recognizes the family as the child’s most significant and persistent source of support, playing a pivotal role in ensuring both family satisfaction and the effectiveness of the services provided [25]. Therefore, effective communication with the families of children with NDDs is considered to support their participation by addressing their interpretation of family participation in the context of FCS.

A further point to consider is that even when the parents of children with NDDs used participation strategies against stigma, the children with NDDs had lower participation scores and worse quality of life than typically developing children. Therefore, based on the results of the correspondence analysis of the participation strategies, we must consider how to support them. Among the participation-based approaches currently in use, the key to the foundation of an evidence-supported approach is how to discover “effective strategies”. These strategies offer coaching and provide support in the child’s natural, everyday environment [2,4,5,6,26].

Furthermore, the results of our study reveal that these strategies are setting-specific. In addition, the first component of the correspondence analysis revealed that these participation strategies were divided into the school/community setting and home setting. This finding suggests that the difference between the public setting (school and community) and the private setting (home) affects the features of this strategy. Schools and communities offer specific opportunities for children to spend time apart from their parents. According to the results of this study, they used strategies that enhanced resources for participation, such as friends, neighbors, and teachers. They also used strategies to pre-arrange the prospects for the children. Therefore, social support is necessary, including reducing stigma for participation in public settings, which is more interpersonally complex. Reference should be made to the model for a paradigm shift in pediatric rehabilitation, which has been proposed in recent years to address these issues [1,27,28]. Several layers related to participation strategies must be considered to meet the various needs of children and their parents. These strategies should cover the micro (e.g., client/family, service providers), meso (e.g., administrators within organizations such as rehabilitation centers, hospitals, and schools), and macro levels (e.g., local and governmental policy, regulatory bodies) [1]. The results of this study provide a perspective on how to promote these practices.

This study has limitations that should be considered. The study population comprised children/parents using a single pediatric rehabilitation hospital. Therefore, its generalizability is limited to some extent. Additionally, the participation was heavily influenced by the environment. Regarding the data comparison of PEM-CY, it is necessary to examine cultural differences. Thus, future research should be conducted in various regions of Japan. Furthermore, this study used cross-sectional data and, thus, could not capture time factors or changes. Future research should take these limitations into account.

## 5. Conclusions

This study analyzed the participation strategies of the parents of children with NDDs. The results suggest the use of participation strategies to dissolve stigma, especially in schools and communities. In addition, the strategies used in each setting were specific, suggesting the need to discover and use effective strategies in the child’s natural environment to enable participation. In other words, these results again suggest the importance of FCS and social approaches, as well as individualized approaches, in supporting the participation of children with NDDs and their parents. In addition, data on the participation strategies obtained in this study reflect the proximity of the clients and may provide clues for future approaches. In the future, we aim to examine participation over time and from more diverse backgrounds (i.e., age, region, and economic status).

## Figures and Tables

**Table 1:** Participant’s characteristics.

Child Demographics	Total
Total, *n*	92
Sex, *n* (%)	
Male	65 (71)
Female	27 (29)
Age (years), mean (SD)	7.6 (1.6)
6 years, *n* (%)	33 (36)
7 years, *n* (%)	17 (18)
8 years, *n* (%)	13 (14)
9 years, *n* (%)	12 (13)
10 years, *n* (%)	11 (12)
11 years, *n* (%)	6 (7)
Type of classroom, *n* (%)	
Regular classes	36 (39)
Special support services in resource rooms	12 (13)
Special support classes	29 (32)
Special support schools	15 (16)
Major diagnosis, *n* (%)	
Autism Spectrum Disorder	28 (30)
Attention-Deficit/Hyperactivity Disorder	7 (8)
Developmental coordination disorder	22 (24)
Specific learning disorder	14 (15)
Intellectual disorder	21 (23)
KINDL ^1^ scores as a measure of QOL, mean (SD)	
Total subscale score	69.3 (10.6)
Physical	82.7 (13.2)
Emotional	77.5 (14.2)
Self-esteem	57.7 (20.4)
Family	65.7 (13.4)
Friends	63.7 (18.1)
School	68.7 (17.3)

^1^ Kid- & Kiddo-KINDL Parents’ Version.

**Table 2:** PEM-CY scores.

Measure	Setting	Total Mean (SD)
Frequency (8-point scale)	Home	5.58 (0.85)
	School	3.97 (1.53)
	Community	1.97 (0.96)
Involvement (5-point scale)	Home	3.90 (0.70)
	School	3.51 (1.20)
	Community	3.18 (1.20)
Desire for change (percentage)	Home	64 (28)
	School	61 (36)
	Community	56 (31)
Overall environmental support (percentage)	Home	80 (12)
	School	84 (12)
	Community	81 (12)

## Data Availability

The data presented in this study are available on request from the corresponding author. The data are not publicly available due to privacy and ethical restrictions.
